# Analysis of the functional diagnosis and Tobin index for failure in weaning from mechanical ventilation

**DOI:** 10.1186/cc14698

**Published:** 2015-09-28

**Authors:** Maíra J Maturana, Fabiana RF Arnone, Gabriela M Lucin, Larissa Domanski, Luiz Alberto M Knaut, Tais G de Matos, Esperidião E Aquim

**Affiliations:** 1 1-Faculdade Inspirar, Cutiriba-PR, São Francisco, Curitiba, SP, Brazil

## Introduction

There is a big divergence regarding the indexes that show whether an extubation process is successful or not. Regardless of this, the spontaneous breathing trial is the most recommended for that aim.

## Objective

To identify the reason for failure in the weaning process and to relate it to the Tobin index.

## Methods

Experimental study of patients admitted to the ICUs of the Tabalhador, Vita Curitiba, Vita Batel Hospitals and the Neurology Institute of Curitiba (INC), in the city of Curitiba, between April and December 2014. Thirty spontaneous breathing trials were taken with pressure support (PS) of 7, positive end-expiratory pressure (PEEP) of 5 cmH_2_O and inspired oxygen fraction = 0.4 for 30 minutes in 17 men and 13 women, with an average age of 51.4 (±24.2), who have been intubated for more than 24 hours in mechanical ventilation and are able to go through the mechanical ventilation weaning process. Upon failure of the SBT, the reason for the lack of success should be classified through functional diagnosis, divided into increase of resistance, characterized by decrease in respiratory rate (FR35), decrease of chest expansibility, pulmonary auscultation with minor vesicular groan diminished or ceased, noises from crackling or small bubbling and usage of accessory inspiratory muscle formation or still alteration of functional reserve. After the identification of the functional diagnosis, he Tobin index was calculated with zero PS and PEEP maintained at 5 cmH_2_O.

## Results

**Figure 1:**
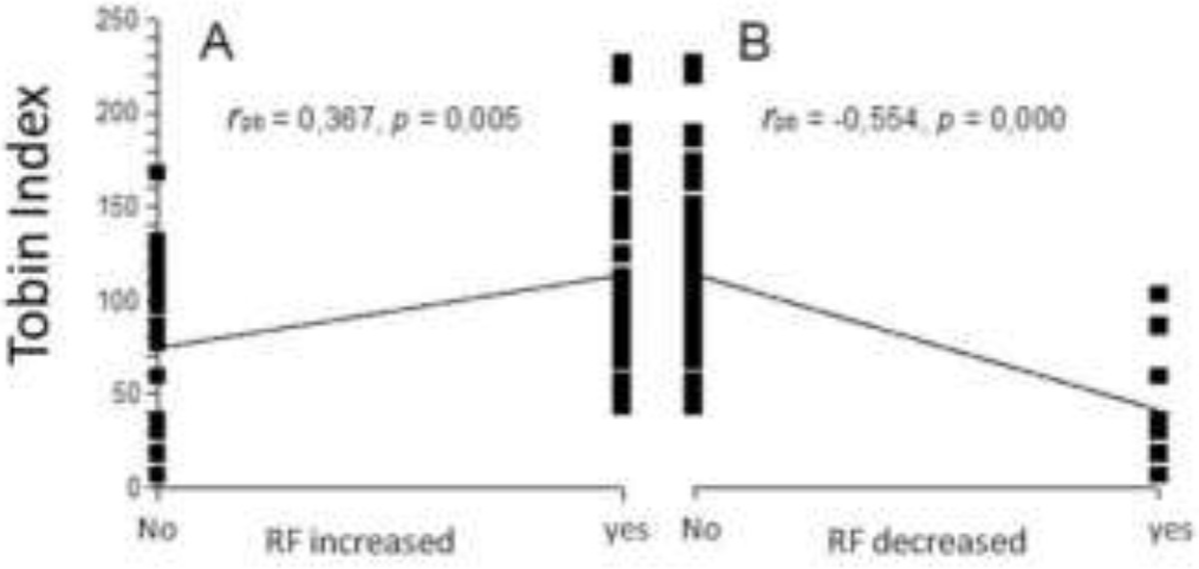
A Relationship between the Tobin index and the increase of respiratory frequency (RF). B Relationship between the Tobin index and the reduction of respiratory frequency (RF)

Of the 30 TER 14 evolved with failure, where FR was predictive of failure in the weaning process and when related to the Tobin index (Figure 1). Increased FR (Figure 1A) showed a slightly positive correlation (*r*_pb _= 0.367; *p *= 0.005), whilst decreased FR (Figure 1B) showed a slightly negative correlation (*r*_pb _= -0.554; *p *= 0.000).

## Conclusion

The functional diagnosis may be an auxiliary predictive indicator for the failure in mechanical ventilation weaning when related to the Tobin index.
